# Distribution of High-Sensitivity C-Reactive Protein and Its Relationship with Other Cardiovascular Risk Factors in the Middle-Aged Chinese Population

**DOI:** 10.3390/ijerph13090872

**Published:** 2016-08-31

**Authors:** Zengwu Wang, Xin Wang, Zuo Chen, Linfeng Zhang, Manlu Zhu

**Affiliations:** Division of Prevention and Community Health, National Center for Cardiovascular Disease, Fuwai Hospital, Peking Union Medical College & Chinese Academy of Medical Sciences, No. 167, Beilishi Road, Xicheng District, Beijing 100037, China; wangxinfw@163.com (X.W.); chenzuo007@sina.com (Z.C.); lf_zh@sina.com (L.Z.); manluzhu@126.com (M.Z.)

**Keywords:** high-sensitivity C-reactive protein, cardiovascular disease, risk factor, blood pressure

## Abstract

*Background:* An increased concentration of high-sensitivity C-reactive protein (hs-CRP) indicates risk for cardiovascular disease (CVD). Because the available data is limited, a cross-sectional survey was conducted in 2009–2010 to describe hs-CRP distribution and its relationship with established CVD risk factors. *Methods:* A population-based sample of adults aged 35 to 64 years (*n* = 14,046) was taken from 12 research populations across China. Demographic and clinical characteristics were recorded, and hs-CRP measured. Pearson’s and Kendall’s tau-b correlation coefficient, and multiple regression analyses were used to test the relationship between hs-CRP and other CVD risk factors. *Results:* For 8389 (4412 females) eligible participants, hs-CRP was 1.89 ± 4.37 mg/L (median (25th, 75th): 0.80 (0.40, 1.80)), and increased with age, BP, glucose, and BMI (*p <* 0.05), males had significantly higher hs-CRP than females (2.07 (4.89) vs. 1.73 (3.83), *p <* 0.001). About 24.3% had the hs-CRP concentrations more than the top quartile (25.8% in males, 22.9% in females), 12.3% (13.3% in males, 11.5% in females) >3 mg/L. There was a significant positive correlation of quartiles of hs-CRP concentrations with age, SBP, DBP, glucose level, BMI, LDL-C/HDL-C ratio, and LDL-C/total cholesterol ratio (*p* < 0.001). The elevated hs-CRP (>1.80 mg/L) related positively with age, LDL-C, BP, glucose, BMI, and living north and negatively with HDL-C/TC, LDL-C/TC, TC independently (*p <* 0.05). For subjects with coexisting hypertension, diabetes, high cholesterol, and obesity, about 63.0% were in the top quartile of hs-CRP concentrations. *Conclusions:* Hs-CRP was associated with most of the known CVD risk factors. Measurement of hs-CRP may provide a more comprehensive view of the patient’s overall risk profile in the Chinese population.

## 1. Introduction

Despite improved control of risk factors associated with cardiovascular disease (CVD) [1,2], it remains a leading cause of mortality worldwide, particularly in Asian countries such as China [1,3,4]. According to the recommendations of the American Heart Association (AHA), measurement of the levels of a known chronic inflammatory marker, high-sensitivity C-reactive protein (hs-CRP), as an adjunct to the established risk factors, is considered optimal for assessment of cardiovascular risk [5].

Utilizing CRP concentrations in addition to standard risk factors such as age, sex, smoking status, blood pressure, history of diabetes, and total cholesterol level has provided meaningful predictive value to future CV risk prediction in, primarily, North American and European patients without a history of CVD [6]. In the large-scale, multinational European Study on Cardiovascular Risk Prevention and Management in Usual Daily Practice (EURIKA), the prevalence of high CRP concentrations (38.2% patients with CRP concentrations ≥3 mg/L and 54.1% patients with CRP concentrations ≥2 mg/L) in patients considered to be at intermediate risk by traditional risk-estimation systems indicated that the use of CRP concentrations was more sensitive in identifying patients who were at a higher risk of CVD [7]. In this context, CRP has also been considered a stronger predictor of cardiovascular events than the conventional risk factor, low-density lipoprotein cholesterol (LDL-C); additionally, it has shown a consistent long-term association with cardiovascular risk, independent of various patient subgroups [7,8]. Therefore, examining hs-CRP concentrations may aid in primary prevention of CVD, especially in patients who are otherwise not identified by lipid assessment alone [9].

Although there is considerable evidence associating hs-CRP with CVD risk [6,7,8,10,11,12], the clinical applicability of hs-CRP remains controversial. Seo and colleagues [13] reported a significant positive correlation between hs-CRP and various cardiovascular risk factors in Korean patients with CVD or diabetes mellitus. Gomez-Marcos reported that hs-CRP was related to arterial stiffness in hypertensive patients [14]. In China, hs-CRP concentrations have been closely associated with cardiovascular risk factors such as hypertension [10] and arterial stiffness [15], and with coronary artery disease [16,17], however, these study participants were selected from worksites [10] or health examinations [15], or from one or two study populations [17,18].

As known, CRP concentrations are variable with ethnic status [19], and are also different between genders from Asian people to Western people [20,21]. With respect to the high risk cut point of CRP (3 mg/L) [22], it may be remarkably higher for Asian people [12,18,21]. Currently, the available large-scale population-based data on hs-CRP across China are insufficient to conclusively promote its use as a marker for screening of cardiovascular risk. Therefore, we sought to explore the distribution of hs-CRP and its relationship with other cardiovascular risk factors in the middle-aged general population without a history of CVD in 12 study populations across China.

## 2. Methods

The detailed methods of the survey have been described previously [23]. Briefly, this cross-sectional survey for evaluating CVD risk factors was conducted in 2009–2010. Twelve research populations, including southern and northern, urban and rural in different parts of China, were selected based on the economic and social development level and the basis of previous research. Of the 14,046 individuals invited, 11,623 participated in the study (response rate = 82.75%). A total of 8389 individuals without prior CVD were subjected to further analysis after excluding patients with chronic inflammatory diseases such as malignancies, hepatic or renal diseases, and acute infectious diseases, as these patients were already considered to have high hs-CRP concentrations. Approximately 1000 inhabitants, comprised of equal proportions of males and females in the age range of 35 to 64 years, were recruited from each population using a random-cluster-sampling method. The study protocol was approved by the institutional review board and ethics review committee of Fuwai Hospital as well as each participating center. All participants provided written informed consent to participate in the study.

Demographic and clinical data, including age, smoking status, body mass index (BMI), and blood pressure (BP), were collected from the participants in accordance with a standard protocol. Blood was drawn in the morning after an overnight fast. All blood samples were processed at the field centers and immediately frozen, and then shipped to the designated central clinical laboratories (Beijing CIC Clinical Laboratory, Beijing, China) for assessment.

Fasting glucose, total cholesterol, high-density lipoprotein cholesterol (HDL-C), triglyceride, and uric acid (UA) were measured using enzymatic techniques on a HITACHI 7080 autoanalyzer (Hitachi, Ltd., Tokyo, Japan). For individuals with triglyceride levels ≥400 mg/dL, LDL-C was calculated indirectly using the Friedewald formula [24]; LDL-C was tested directly for individuals with triglyceride levels <400 mg/dL; non-HDL-C equal TC minus HDL-C. Estimation of hs-CRP was done using transmission turbidimetry (Advia 2400 autoanalyzer, Siemens, Munich, Germany).

The BP measurement recorded for analysis was an average of three readings taken using a conventional sphygmomanometer (mercury) from the right arm at the level of the heart after the participant had been seated for 5 min, with a 30-s gap between each measurement. Hypertension was defined as systolic BP (SBP) ≥ 140 mmHg, diastolic BP (DBP) ≥90 mmHg, and/or ongoing treatment for hypertension. BMI was defined as the ratio of weight to height squared (kg/m^2^). Being overweight was defined as BMI ≥ 24 and < 28 kg/m^2^ and obesity as BMI ≥ 28 kg/m^2^ according to the Chinese guidelines [25]. Patients were considered to have diabetes if their fasting glucose level was ≥7.0 mmol/L (126 mg/dL), or if they were undergoing treatment with insulin or oral hypoglycemic agents. Participants were considered smokers if they consumed more than 1 cigarette a day for at least 1 year or had used at least 20 packets of cigarettes (equivalent to 0.5 kg of tobacco leaves) in their lifetime. The individual cardiovascular risk factors considered in this study were age (males ≥ 45 years and females ≥ 55 years), LDL-C > 2.60 mmol/L (100 mg/dL), HDL-C < 1.04 mmol/L (40 mg/dL), high cholesterol (total cholesterol ≥6.22 mmol/L (240 mg/dL)), smoking, and the presence of hypertension, diabetes, overweight, or obesity.

All statistical analyses were performed using the SAS version 9.2 (SAS Institute Inc., Cary, NC, USA). Continuous variables were presented as the mean (standard deviation (SD)). Since the hs-CRP concentrations were skewed to left, median (25th percentile, 75th percentile) (M (25, 75)) were also used. Categorical variables were described as counts and percentages. Differences in continuous variables were assessed using two-tailed *t*-tests for two groups, and using ANOVA for more than two groups; differences in categorical variables were identified using the chi-square and Fisher’s exact tests. Analysis was performed based on ranges of hs-CRP quartiles (0 to Q1, Q1 to Q2, Q2 to Q3, and Q3 to Q4) to evaluate the variation in risk factors; the Pearson’s correlation coefficient and Kendall’s tau-b correlation coefficient were used to test the relationship among the continuous and categorical variables, respectively. The top quartile value of hs-CRP (1.80 mg/L) was as the cut point for dichotomous variable, hs-CRP values more than the top quartile values were defined as elevated hs-CRP. Multiple regression analyses (enter regression model) were also used to examine the independent associations between hs-CRP and other risk factors. Crude and adjusted odds ratios (ORs) and 95% confidence intervals (CIs) were calculated for other risk factors. A *p*-value < 0.05 was considered statistically significant.

## 3. Results

The baseline characteristics of 3977 males and 4412 females included in the analysis are listed in Table 1. Overall, the average age was 49.46 ± 8.06 years, and a large proportion of males were smokers (64.2%). Hypertension and diabetes were found in approximately 39.0% and 9.3% of individuals, respectively. Significant gender differences were observed in the baseline characteristics except LDL-C, HDL-C level and the rate of high cholesterol, BMI, urban/rural, and north/south (*p <* 0.05).

Overall, the average concentrations of hs-CRP was 1.89 ± 4.37 mg/L (M (25, 75): 0.80 (0.40, 1.80)), and increased with age, BP, glucose, and BMI (*p <* 0.05), males had significantly higher hs-CRP than females (2.07 (4.89) vs. 1.73 (3.83), *p <* 0.001). Out of them, 24.3% had the hs-CRP concentrations more than top quartile (25.8% in males, 22.9% in females), 12.3% (13.3% in males, 11.5% in females) >3 mg/L. Participants with low HDL-C, as well as smokers, and live in north area, had higher hs-CRP concentrations than their counterparts (*p <* 0.05, Table 2). Difference between genders was no significant for the positive participant with high LDL-C, low HDL-C, high cholesterol, smokers, drinking, and live in north area (Table 2).

There was a significant positive correlation of quartiles of hs-CRP concentrations with age, SBP, DBP, glucose level, BMI, LDL-C/HDL-C ratio, and LDL-C/total cholesterol ratio (*p* < 0.001; Table 3). Additionally, a significant negative correlation was observed for hs-CRP concentrations with HDL-C and the HDL-C/total cholesterol ratio (*p* < 0.001). The proportion of patients with hypertension, diabetes, or high cholesterol increased with the increase of quartile grade of hs-CRP concentrations (*p* < 0.001).

Further, multivariate logistic regression model was used to analyze the independent association between hs-CRP and other known CV risk factors (Table 4). The prevalence of elevated hs-CRP related positively with age, education, smoking, LDL-C, BP, glucose, BMI, and living north and negatively with LDL-C/TC, TC (*p <* 0.05); and did not relate with drinking, HDL-C/TC, LDL-C/HDL-C, non-HDL-C, living urban/rural (*p* > 0.05).

The association between CVD risk factors with hs-CRP remained in males and females (Table 5). However, elevated hs-CRP related with BP, education for males, with age, TC, LDL-C for females, and with smoking, glucose, BMI, and living region of north for both gender (*p <* 0.05).

**Figure 1:**
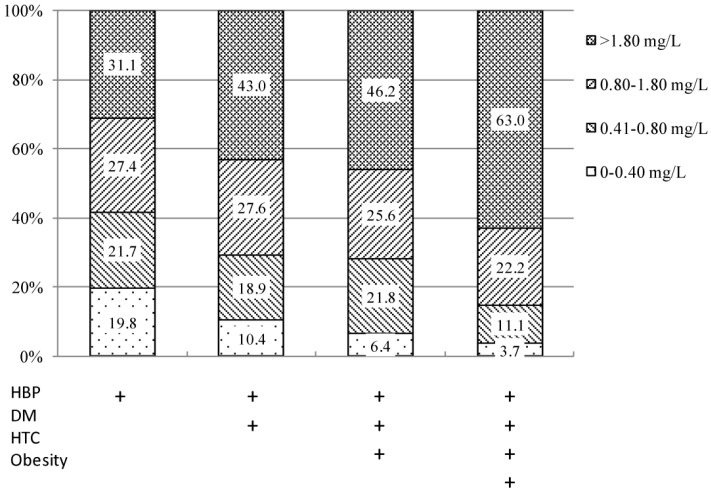
Proportion of hs-CRP in patients with different risk factor combinations; Hs-CRP, high-sensitivity C-reactive protein; HBP, high blood pressure; DM, diabetes mellitus; HTC, high total cholesterol.

Moreover, for subjects with hypertension, about 31.1% were in the top quartile of hs-CRP concentrations; for subjects with coexisting hypertension, diabetes, high cholesterol, and obesity, 63.0% in the top quartile (Figure 1).

## 4. Discussion

In this cross-sectional population-based study in China, hs-CRP was investigated, and its association with other established risk factors was evaluated. In particular, aging, high levels of LDL-C, as well as BP, glucose, BMI were independently associated with a higher hs-CRP concentrations, especially in individuals with coexisting diabetes, hypertension, high cholesterol, and obesity, although, the differences remained between genders. Overall, the mean hs-CRP concentrations observed in the present study (1.89 mg/L) were higher than those observed in the Korean population (1.32 mg/L) [13], although participants in the Korean research had CVD or diabetes mellitus.

According to the criteria of ACCF/AHA [22], if CRP concentrations >3 mg/L was with “high risk”, the proportion was 12.3% in our study, lower than the top quartile proportion (24.3%), but higher than Zhao’s investigation in Shanghai, China [18]; and also higher than that of K.C. Sung’s study [12] both males (13.3% vs. 8.6%) and females (11.5% vs. 6.2%). In general, the CRP concentrations in Asia people is lower than that in western people, it is still associated with CVD risk factors remarkably, and appears to independently predict CVD and all-cause mortality [12]. Measurement of hs-CRP may provide a more comprehensive view of the patient’s overall risk profile in the Chinese population, or Asian people; although measurement of CRP is not recommended by ACCF/AHA for cardiovascular risk assessment in low-risk men younger than 50 years of age or women 60 years of age or younger [5].

Higher hs-CRP concentrations in males compared with females was also observed in our study, but the condition is reversed in western people [26]. It is noteworthy that several variables can affect hs-CRP. For example, Dar and colleagues [27] pointed out that although high SBP and DBP are associated with high hs-CRP concentrations, a prolonged history of hypertension could lower hs-CRP concentrations. Similarly, our study witnessed higher concentrations of hs-CRP with increasing SBP and DBP. It would be interesting to observe temporal trends in hs-CRP in hypertensive patients, and this could be considered for future longitudinal studies of Chinese patients.

Identifying individuals at high risk of CVD is a major challenge for preventing CVD, and biomarker-based risk prediction is rapidly evolving. Previous studies have documented a similar association between hs-CRP and CVD risk factors. In studies conducted in China, hs-CRP has been associated with HDL-C, BP, and lifestyle variables in the general population [17,28], as well as several CVD risk factors in hypertensive patients [10,15]. In a nationwide study among Korean adults, correlation of plasma hs-CRP concentrations and cardiovascular risk in the Korean population (CALLISTO) found enhanced hs-CRP concentrations with older age, male gender, high blood pressure or hypertension, low HDL-C, and high LDL-C, as well as correlation of hs-CRP concentrations with BMI, LDL-C to total cholesterol ratio, and LDL-C to HDL-C ratio. In agreement with the published study, we also observed that males had higher hs-CRP concentrations than females, which may be due to more males having high levels of SBP, DBP, and blood glucose. As the results showed that people with hypertension, diabetes or had higher hs-CRP concentrations; if coexisting with hypertension, diabetes, high cholesterol, and obesity, 63.0% of them were in the top quartile of hs-CRP. So, high risk participants normally were accompanied with higher hs-CRP.

Our results showed that living north had higher hs-CRP concentrations compared with living south, whereby, there was no significant difference between living rural or urban area. Another study reported that hs-CRP was not a risk factor among rural adults older than age 50 in Korea [26]. However, this could be attributable to differences in lifestyle, daily activities, and dietary habits between different populations. It also implied that the north-south differences had much stronger influence on hs-CRP than that of the rural-urban difference in China.

Alcohol consumption was not found to be associated with hs-CRP in the present study, whereas, smoking, known CVD risk factor, was related to higher hs-CRP. Further analysis showed, smoking increases the risk of elevated hs-CRP in women (OR, 1.41; 95% CI, 1.06, 1.89) than in men (OR, 1.22; 95% CI, 1.04, 1.44). One of the reasons may be that a large part of men are smokers. Although there is controversial, Chien and colleagues developed a clinical model for prevention of stroke in a Chinese population; they noticed that smoking status, as a variable, did not add any significant value to the prediction of stroke in the Chinese population [29].

Seo and colleagues found that LDL-C > 100 mg/dL was associated with a lower median hs-CRP concentrations in Korean adults with CVD or diabetes; however, multivariate regression analysis showed that neither LDL-C nor smoking were independently correlated with hs-CRP concentrations [13]. In our study, LDL-C showed significant correlation with the inflammatory markers. But non-HDL-C, considered as a more powerful predictor of future CVD event than LDL-C, was not independently correlated with hs-CRP concentrations after adjustment other risk factors, the results were similar by genders. Hs-CRP is a known marker of inflammation due to oxidative stress. Hyperlipidemia may not immediately increase hs-CRP concentrations because oxidative stress occurs following accumulation of cholesterol in the lipid pool. The oxidative stress generates oxysterol, which is highly toxic and enhances inflammation, followed by the onset of atherosclerosis [30].

The study had some limitations. Its cross-sectional and observational design did not allow the evaluation of the temporal relationship between future cardiovascular events and hs-CRP concentrations. Therefore, the current findings need to be confirmed by future adequately powered prospective trials and long-term studies. Despite the use of random cluster sampling, there may have been selection bias due to unresponsive participants. In addition, lifestyle variables such as exercise or diet were not included. As some of these lifestyle parameters have been shown to be independently related to CVD events in Chinese populations [31], their association with hs-CRP could potentially be examined in future studies. Another limitation emanated from the inability to exclude the possibility that some participants had experienced undiagnosed CVD events that could have resulted in higher hs-CRP concentrations.

## 5. Conclusions

This large-scale, population based study indicated that to investigate the hs-CRP concentrations was higher comparing with the past and Korea, but lower than in western. There was significant association between hs-CRP concentrations and cardiovascular risk factors in the general Chinese population. Incorporating hs-CRP biomarker in clinical assessment may provide a more comprehensive view of the patient’s overall risk profile and could improve outcomes for primary prevention of CVD.

## Figures and Tables

**Table 1:** Baseline clinical and laboratory characteristics.

Characteristics	Total (*n* = 8389)	Male (*n* = 3977)	Female (*n* = 4412)	*p*-Value
Age, years	49.46 (8.06)	49.77 (8.13)	49.18 (7.98)	0.001
Education, *n* (%)				<0.001
Below high school	1732 (43.6)	2272 (51.5)	4004 (47.7)	
High school or above	2244 (56.4)	2140 (48.5)	4384 (52.3)	
Smoking, *n* (%)	2877 (34.3)	2554 (64.2)	323 (7.3)	0.001
Drinking, *n* (%)	1835 (21.9)	1640 (41.2)	195 (4.4)	0.001
SBP, mmHg	128.97 (20.52)	130.20 (19.82)	127.86 (21.07)	<0.001
DBP, mmHg	82.44 (11.65)	83.93 (11.82)	81.10 (11.33)	<0.001
Glucose, mmol/L ^a^	5.73 (1.50)	5.80 (1.57)	5.67 (1.42)	<0.001
BMI, kg/m^2^	24.37 (3.61)	24.20 (3.55)	24.53 (3.67)	0.001
Total cholesterol, mmol/L	4.80 (0.93)	4.78 (0.92)	4.82 (0.94)	<0.001
LDL-C, mmol/L	2.75 (0.77)	2.75 (0.77)	2.75 (0.78)	0.083
HDL-C, mmol/L	1.39 (0.32)	1.34 (0.32)	1.43 (0.31)	0.755
Triglycerides, mmol/L	1.61 (1.13)	1.70 (1.25)	1.52 (0.99)	<0.001
Non-HDL-C, mmol/L	3.41 (0.93)	3.44 (0.93)	3.39 (0.93)	0.010
Uric acid, µmol/L	264.45 (77.85)	304.64 (74.94)	228.18 (60.73)	<0.001
High cholesterol, *n* (%)	618 (7.4)	282 (7.1)	336 (7.6)	0.366
Low HDL-C, *n* (%)	922 (11.0)	597 (15.0)	325 (7.4)	<0.001
Hypertension, *n* (%)	3272 (39.0)	1699 (42.7)	1573 (35.7)	<0.001
Diabetes, *n* (%) ^a^	781 (9.3)	412 (10.4)	369 (8.4)	0.002
BMI, kg/m^2^, *n* (%)				
<24	3721 (44.4)	1795 (45.1)	1926 (43.7)	0.129
≥24, <28	3104 (37.0)	1475 (37.1)	1629 (36.9)	
≥28	1564 (18.6)	707 (17.8)	857 (19.4)	
Urban, *n* (%)	965 (24.3)	1124 (25.5)	2089 (24.9)	0.129
Rural, *n* (%)	3012 (75.7)	3288 (74.5)	6300 (75.1)	
North, *n* (%)	2070 (52.0)	2333 (52.9)	4403 (52.5)	0.200
South, *n* (%)	1907 (48.0)	2079 (47.1)	3986 (47.5)	

Data are presented as mean (standard deviation) unless indicated otherwise. Abbreviations: BMI, body mass index; DBP, diastolic blood pressure; HDL-C, high-density lipoprotein cholesterol; LDL-C, low-density lipoprotein cholesterol; SBP, systolic blood pressure; ^a^ no data for eleven subjects.

**Table 2:** Distribution of hs-CRP by individual risk factors.

Item	Total			*p*-Value ^a^	Male			Female			*p*-Value ^b^
	N	Mean (SD)	M (25, 75)		N	Mean (SD)	M (25, 75)	N	Mean (SD)	M (25, 75)	
Total	8389	1.89 (4.37)	0.80 (0.40, 1.80)	--	3977	2.07 (4.89)	0.90 (0.50, 1.9)	4412	1.73 (3.83)	0.80 (0.30, 1.70)	<0.001
Age, year				<0.001							
<45	2646	1.56 (3.88) ^a^	0.60 (0.30, 1.40)		1201	1.81 (4.35)	0.80 (0.40, 1.70)	1445	1.36 (3.44)	0.50 (0.30, 1.20)	0.003
46–54	3130	1.91 (4.65)	0.80 (0.40, 1.80)		1488	2.12 (5.25)	0.90 (0.50, 1.90)	1642	1.72 (4.02)	0.80 (0.40, 1.60)	0.015
≥55	2613	2.20 (4.45)	1.00 (0.50, 2.10)		1288	2.26 (4.93)	1.00 (0.50, 2.00)	1325	2.15 (3.94)	1.10 (0.50, 2.20)	0.550
LDL-C > 2.60 mmol/L				0.421							
Yes	416	2.06 (3.24)	1.20 (0.60, 2.30)		185	2.36 (3.93)	1.20 (0.70, 2.30)	231	1.82 (2.54)	1.10 (0.60, 2.20)	0.095
No	7973	1.88 (4.42)	0.80 (0.40, 1.80)		3792	2.06 (4.93)	0.90 (0.50, 1.90)	4181	1.73 (3.89)	0.70 (0.30, 1.70)	0.001
HDL-C < 1.04 mmol/L				<0.001							
Yes	922	2.86 (5.99) ^a^	1.20 (0.60, 2.50)		597	2.89 (5.85)	1.30 (0.70, 2.50)	325	2.82 (6.26)	1.10 (0.50, 2.50)	0.871
No	7467	1.77 (4.11)	0.80 (0.40, 1.70)		3380	1.92 (4.69)	0.80 (0.40, 1.80)	4087	1.64 (3.56)	0.70 (0.30, 1.70)	0.003
TC ≥ 6.22 mmol/L				0.298							
Yes	618	2.07 (3.30)	1.20 (0.68, 2.30)		282	2.27 (3.84)	1.25 (0.70, 2.40)	336	1.90 (2.76)	1.20 (0.60, 2.20)	0.157
No	7771	1.88 (4.44)	0.80 (0.40, 1.80)		3695	2.06 (4.96)	0.90 (0.40, 1.90)	4076	1.72 (3.90)	0.70 (0.30, 1.60)	0.001
Smoking				0.020							
Yes	2877	2.05 (4.61) ^a^	0.90 (0.50, 1.90)		2554	1.99 (4.45)	0.90 (0.50, 1.90)	323	2.51 (5.71)	0.90 (0.40, 2.10)	0.057
No	5512	1.81 (4.23)	0.80 (0.40, 1.70)		1423	2.22 (5.59)	0.90 (0.50, 1.90)	4089	1.67 (3.63)	0.70 (0.30, 1.70)	0.001
Drinking				0.698							
Yes	1835	1.86 (4.58)	0.80 (0.40, 1.70)		1640	1.93 (4.81)	0.80 (0.40, 1.70)	195	1.25 (1.63)	0.70 (0.30, 1.60)	0.051
No	6554	1.90 (4.30)	0.80 (0.40, 1.80)		2337	2.17 (4.94)	1.00 (0.50, 2.00)	4217	1.75 (3.90)	0.80 (0.40, 1.70)	0.001
Blood pressure, mmHg				<0.001							
<140/90	5473	1.76 (4.59) ^a^	0.70 (0.30, 1.50)		2433	2.02 (5.32)	0.80 (0.40, 1.70)	3040	1.55 (3.90)	0.60 (0.30, 1.40)	0.001
≥140/90, <160/100	1861	2.10 (3.84)	1.00 (0.50, 2.20)		969	1.99 (3.61)	1.00 (0.50, 2.00)	892	2.21 (4.07)	1.10 (0.50, 2.30)	0.224
≥160/100, <180/110	742	2.07 (3.76)	1.10 (0.60, 2.20)		404	2.23 (4.53)	1.10 (0.60, 2.20)	338	1.89 (2.52)	1.10 (0.60, 2.30)	0.229
≥180/110	313	2.56 (4.56)	1.40 (0.60, 2.85)		171	2.88 (5.54)	1.50 (0.60, 3.00)	142	2.18 (2.96)	1.30 (0.60, 2.63)	0.180
Glucose, mmol/L ^c^				<0.001							
<6.1	6720	1.70 (3.92) ^a^	0.80 (0.40, 1.60)		3095	1.94 (4.74)	0.80 (0.40, 1.80)	3625	1.50 (3.03)	0.70 (0.30, 1.50)	0.001
≥6.1, <7.0	937	2.22 (4.94)	1.10 (0.50, 2.20)		490	1.94 (3.28)	1.10 (0.60, 2.00)	447	2.53 (6.26)	1.10 (0.50, 2.50)	0.006
≥7.0	721	3.18 (6.65)	1.50 (0.70, 2.80)		385	3.19 (7.07)	1.40 (0.70, 2.50)	336	3.17 (6.14)	1.60 (0.70, 3.00)	0.974
BMI, kg/m^2^				<0.001							
<24	3721	1.62 (4.59) ^a^	0.60 (0.30, 1.20)		1795	2.00 (5.66)	0.70 (0.40, 1.50)	1926	1.28 (3.26)	0.50 (0.20, 1.10)	0.001
≥24, <28	3104	1.90 (4.12)	0.90 (0.50, 1.80)		1475	2.00 (3.94)	1.00 (0.50, 1.90)	1629	1.81 (4.28)	0.80 (0.40, 1.70)	0.201
≥28	1564	2.52 (4.23)	1.50 (0.80, 2.70)		707	2.42 (4.55)	1.40 (0.80, 2.40)	857	2.61 (3.94)	1.60 (0.90, 3.00)	0.367
Urban/Rural				0.925							
Urban	2089	1.90 (4.11)	0.90 (0.50, 1.90)		965	2.06 (4.66)	1.00 (0.50, 1.90)	1124	1.76 (3.56)	0.90 (0.40, 1.90)	0.096
Rural	300	1.89 (4.45)	0.80 (0.40, 1.80)		3012	2.07 (4.96)	0.90 (0.40, 1.90)	3288	1.72 (3.91)	0.70 (0.30, 1.60)	0.002
South/North				<0.0001							
North	4403	2.17 (4.68)	1.00 (0.50, 2.10)		2070	2.30 (5.24)	1.00 (0.50, 2.10)	2333	2.06 (4.11)	0.90 (0.40, 2.10)	0.097
South	3986	1.58 (3.97)	0.70 (0.30, 1.50)		1907	1.83 (4.47)	0.80 (0.40, 1.60)	2079	1.36 (3.44)	0.60 (0.30, 1.30)	0.001

Variations in numbers are because of missing data. Data are presented as mean (standard deviation) (Mean (SD)) and median (25th percentile, 75th percentile) (M (25, 75)) unless indicated otherwise. Abbreviation: HDL-C, high-density lipoprotein cholesterol; hs-CRP, high-sensitivity C-reactive protein; LDL-C, low-density lipoprotein cholesterol; TC, total cholesterol; BMI, body mass index; SD, standard deviation; ^a^ *p* value within the characteristic; ^b^ *p* value between genders; ^c^ no data for eleven subjects.

**Table 3:** Correlation analyses of different subgroups based on quartile ranges of hs-CRP concentrations and the corresponding cardiovascular risk factors.

Item	Subgroups Based on Quartile Ranges of hs-CRP (mg/L)				Correlation Coefficient ^a^	*p*-Value
	0–0.40 (*n* = 2392)	0.41–0.80 (*n* = 1874)	0.80–1.80 (*n* = 2087)	>1.80 (*n* = 2036)		
Age, years	47.56 (7.97)	49.67 (7.99)	50.03 (8.00)	50.91 (7.85)	0.151	<0.001
SBP, mmHg	123.84 (18.43)	128.38 (20.52)	130.85 (20.33)	133.6 (21.67)	0.178	<0.001
DBP, mmHg	79.64 (10.94)	82.14 (11.45)	83.49 (11.65)	84.93 (11.92)	0.171	<0.001
Glucose, mmol/L	5.43 (1.02)	5.68 (1.37)	5.77 (1.49)	6.09 (1.95)	0.158	<0.001
BMI, kg/m^2^	22.77 (2.90)	24.07 (3.14)	25.17 (3.64)	25.69 (3.98)	0.314	<0.001
LDL-C, mmol/L	2.60 (0.70)	2.77 (0.76)	2.83 (0.80)	2.84 (0.82)	0.119	<0.001
HDL-C, mmol/L	1.48 (0.33)	1.4 (0.30)	1.34 (0.30)	1.31 (0.29)	−0.214	<0.001
TC, mmol/L	4.61 (0.83)	4.79 (0.90)	4.92 (0.95)	4.92 (1.00)	0.132	<0.001
Non-HDL-C, mmol/L	3.12 (0.78)	3.39 (0.91)	3.57 (0.93)	3.61 (1.02)	0. 206	<0.001
Triglycerides, mmol/L	1.29 (0.77)	1.52 (1.08)	1.79 (1.23)	1.83 (1.29)	0.191	<0.001
LDL-C/HDL-C ratio	1.82 (0.58)	2.04 (0.67)	2.18 (0.72)	2.25 (0.74)	0.236	<0.001
LDL-C/TC ratio	0.56 (0.07)	0.57 (0.08)	0.57 (0.08)	0.57 (0.08)	0.065	<0.001
HDL-C/TC ratio	0.33 (0.07)	0.3 (0.07)	0.28 (0.07)	0.27 (0.07)	−0.281	<0.001
Smoking, *n* (%)	710 (29.7)	661 (35.3)	750 (35.9)	756 (37.1)	0.053	<0.001
Hypertension, *n* (%)	648 (27.1)	710 (37.9)	898 (43.0)	1016 (49.9)	0.159	<0.001
Diabetes, *n* (%)	108 (4.5)	151 (8.1)	214 (10.3)	308 (15.2)	0.122	<0.001
High cholesterol, *n* (%)	86 (3.6)	131 (7.0)	197 (9.4)	204 (10.0)	0.088	<0.001

Data are presented as mean (standard deviation) unless indicated otherwise; ^a^ Correlation coefficient was estimated for the correlation analysis between hs-CRP concentrations and cardiovascular risk factors. Kendall’s tau-b correlation coefficient was calculated for smoking, hypertension, diabetes, and high cholesterol. For the remaining risk factors, Pearson’s correlation coefficient was calculated; hs-CRP, high-sensitivity C-reactive protein; SBP, systolic blood pressure; DBP, diastolic blood pressure; BMI, body mass index; LDL-C, low-density lipoprotein cholesterol; HDL-C, high-density lipoprotein cholesterol; TC, total cholesterol.

**Table 4:** Relationship between hs-CRP concentrations and the corresponding cardiovascular risk factors.

Characteristics	β	SE β	OR (95% CI)	*p*-Value
Age, year				
<45 ^a^				
46–54	0.16	0.07	1.17 (1.02, 1.34)	0.02
≥55	0.34	0.07	1.40 (1.21, 1.62)	0.00
High school or above ^a^/Below high school	0.13	0.06	1.14 (1.02, 1.28)	0.02
Non-drinker ^a^/Drinker	−0.08	0.07	0.92 (0.80, 1.06)	0.25
Non-smoker ^a^/Smoker	0.25	0.06	1.28 (1.13, 1.45)	0.00
HDL-C/TC	−0.95	1.41	0.39 (0.02, 6.14)	0.50
LDL-C/HDL-C	0.02	0.17	1.02 (0.74, 1.41)	0.91
LDL-C/TC	−2.09	0.79	0.12 (0.03, 0.58)	0.01
TC	−0.91	0.39	0.40 (0.19, 0.87)	0.02
LDL-C	0.38	0.16	1.46 (1.06, 2.00)	0.02
Non-HDL-C	0.79	0.44	2.20 (0.92, 5.25)	0.07
Blood pressure, mmHg				
<140/90 ^a^				
≥140/90, <160/100	0.22	0.07	1.24 (1.09, 1.41)	0.00
≥160/100, <180/110	0.24	0.09	1.27 (1.06, 1.52)	0.01
≥180/110	0.58	0.13	1.79 (1.39, 2.30)	0.00
Glucose, mmol/L ^b^				
<6.1 ^a^				
≥6.1, <7.0	0.15	0.08	1.17 (0.99, 1.37)	0.06
≥7.0	0.53	0.09	1.69 (1.42, 2.01)	0.00
Body mass index, kg/m^2^				
<24 ^a^				
≥24, <28	0.25	0.06	1.29 (1.13, 1.46)	0.00
≥28	0.80	0.08	2.23 (1.92, 2.59)	0.00
Urban ^a^/Rural	−0.07	0.07	0.94 (0.82, 1.07)	0.34
South ^a^/North	0.35	0.06	1.41 (1.27, 1.58)	0.00

Abbreviations: hs-CRP, high-sensitivity C-reactive protein; OR, odds ratio; CI, confidence interval; LDL-C, low-density lipoprotein cholesterol; HDL-C, high-density lipoprotein cholesterol; TC, total cholesterol; ^a^ Reference group; ^b^ no data for eleven subjects.

**Table 5:** Relationship between hs-CRP concentrations and the corresponding cardiovascular risk factors by gender.

Characteristics	Male	Female
Age, year		
<45 ^a^		
46–54	1.20 (1.00, 1.45)	1.14 (0.93, 1.39) ^b^
≥55	1.21 (1.00, 1.48)	1.60 (1.28, 1.99) ^b^
High school or above ^a^/Below high school	1.23 (1.05, 1.45)	0.99 (0.84, 1.18)
Non-drinker ^a^/Drinker	0.94 (0.79, 1.10)	1.05 (0.71, 1.56)
Non-smoker ^a^/Smoker	1.22 (1.04, 1.44)	1.41 (1.06, 1.89)
HDL-C/TC	0.29 (0.01, 15.36)	0.68 (0.01, 34.56) ^b^
LDL-C/HDL-C	1.19 (0.79, 1.79)	0.85 (0.48, 1.52)
LDL-C/TC	0.19 (0.02, 1.52)	0.10 (0.01, 1.16) ^b^
TC	0.47 (0.16, 1.38)	0.31 (0.10, 0.97) ^b^
LDL-C	1.15 (0.74, 1.79)	1.73 (1.10, 2.74) ^b^
Non-HDL-C	1.97 (0.59, 6.61)	2.92 (0.77, 11.08) ^b^
Blood pressure, mmHg		
<140/90 ^a^		
≥140/90, <160/100	1.13 (0.94, 1.36)	1.37 (1.14, 1.65)
≥160/100, <180/110	1.24 (0.97, 1.59)	1.35 (1.03, 1.76)
≥180/110	2.32 (1.66, 3.24)	1.28 (0.87, 1.90) ^b^
Glucose, mmol/L ^c^		
<6.1 ^a^		
≥6.1, <7.0	1.00 (0.79, 1.26)	1.40 (1.11, 1.77) ^b^
≥7.0	1.53 (1.21, 1.95)	1.94 (1.50, 2.50) ^b^
Body mass index, kg/m^2^		
<24 ^a^		
≥24, <28	1.11 (0.93, 1.32)	1.52 (1.26, 1.83)
≥28	1.49 (1.19, 1.86)	3.25 (2.64, 3.99) ^b^
Urban ^a^/Rural	0.96 (0.80, 1.16)	0.94 (0.77, 1.13)
South ^a^/North	1.37 (1.17, 1.60)	1.41 (1.20, 1.66) ^b^

Data were represented as Odds Ratio (95% confidence interval). Abbreviations: hs-CRP, high-sensitivity C-reactive protein; OR, odds ratio; CI, confidence interval; LDL-C, low-density lipoprotein cholesterol; HDL-C, high-density lipoprotein cholesterol; TC, total cholesterol; ^a^ Reference group; ^b^ *p* value < 0.05 between gender; ^c^ no data for eleven subjects.

## Abbreviations

The following abbreviations are used in this manuscript:

AHA	American Heart Association
ANOVA	analysis of variance
BMI	body mass index
BP	blood pressure
CVD	cardiovascular disease
DBP	diastolic blood pressure
EURIKA	European Study on Cardiovascular Risk Prevention and Management in Usual Daily Practice
HDL-C	high-density lipoprotein cholesterol
hs-CRP	high-sensitivity C-reactive protein
LDL-C	low-density lipoprotein cholesterol
Lp (a)	lipoprotein (a)
SBP	systolic blood pressure
SD	standard deviation
TC	total cholesterol
UA	uric acid
